# Robust growth of avirulent phase II *Coxiella burnetii* in bone marrow-derived murine macrophages

**DOI:** 10.1371/journal.pone.0173528

**Published:** 2017-03-09

**Authors:** Diane C. Cockrell, Carrie M. Long, Shelly J. Robertson, Jeffrey G. Shannon, Heather E. Miller, Lara Myers, Charles L. Larson, Tregei Starr, Paul A. Beare, Robert A. Heinzen

**Affiliations:** 1 *Coxiella* Pathogenesis Section, Laboratory of Bacteriology, Rocky Mountain Laboratories, National Institute of Allergy and Infectious Diseases, National Institutes of Health, Hamilton, Montana, United States of America; 2 Innate Immunity and Pathogenesis Unit, Laboratory of Virology, Rocky Mountain Laboratories, National Institute of Allergy and Infectious Diseases, National Institutes of Health, Hamilton, Montana, United States of America; 3 Plague Section, Laboratory of Zoonotic Pathogens, Rocky Mountain Laboratories, National Institute of Allergy and Infectious Diseases, National Institutes of Health, Hamilton, Montana, United States of America; 4 Retroviral Immunology Section, Laboratory of Persistent Viral Diseases, Rocky Mountain Laboratories, National Institute of Allergy and Infectious Diseases, National Institutes of Health, Hamilton, Montana, United States of America; 5 *Salmonella*-Host Cell Interactions Section, Laboratory of Bacteriology, Rocky Mountain Laboratories, National Institute of Allergy and Infectious Diseases, National Institutes of Health, Hamilton, Montana, United States of America; Texas A&M University Health Sciences Center, UNITED STATES

## Abstract

Published data show that murine bone marrow-derived macrophages (BMDM) restrict growth of avirulent phase II, but not virulent phase I, *Coxiella burnetii*. Growth restriction of phase II bacteria is thought to result from potentiated recognition of pathogen-associated molecular patterns, which leads to production of inhibitory effector molecules. Past studies have used conditioned medium from L-929 murine fibroblasts as a source of macrophage-colony stimulating factor (M-CSF) to promote differentiation of bone marrow-derived myeloid precursors into macrophages. However, uncharacterized components of conditioned medium, such as variable amounts of type I interferons, can affect macrophage activation status and their permissiveness for infection. In the current study, we show that the *C*. *burnetii* Nine Mile phase II (NMII) strain grows robustly in primary macrophages from C57BL/6J mice when bone marrow cells are differentiated with recombinant murine M-CSF (rmM-CSF). Bacteria were readily internalized by BMDM, and replicated within degradative, LAMP1-positive vacuoles to achieve roughly 3 logs of growth over 6 days. Uninfected BMDM did not appreciably express CD38 or Egr2, markers of classically (M1) and alternatively (M2) activated macrophages, respectively, nor did infection change the lack of polarization. In accordance with an M0 phenotype, infected BMDM produced moderate amounts of TNF and nitric oxide. Similar NMII growth results were obtained using C57BL/6J myeloid progenitors immortalized with an estrogen-regulated *Hoxb8* (*ER-Hoxb8*) oncogene. To demonstrate the utility of the ER-Hoxb8 system, myeloid progenitors from natural resistance-associated macrophage protein 1 (Nramp1) C57BL/6J knock-in mice were transduced with *ER-Hoxb8*, and macrophages were derived from immortalized progenitors using rmM-CSF and infected with NMII. No difference in growth was observed when compared to macrophages from wild type mice, indicating depletion of metal ions by the Nramp1 transporter does not negatively impact NMII growth. Results with NMII were recapitulated in primary macrophages where C57BL/6J *Nramp1+* BMDM efficiently killed *Salmonella enterica* serovar Typhimurium. M-CSF differentiated murine macrophages from bone marrow and conditional ER-Hoxb8 myeloid progenitors will be useful *ex vivo* models for studying *Coxiella*-macrophage interactions.

## Introduction

*Coxiella burnetii* is the bacterial cause of human Q fever, an aerosol-transmitted zoonotic infection [1]. This intracellular pathogen has a low human infective dose (~10 microorganisms) and initially targets alveolar macrophages during natural infection [2]. Following receptor-mediated uptake, the nascent *C*. *burnetii*-containing vacuole (CCV) traffics through the canonical endolysosomal pathway to acquire characteristics of a degradative phagolysosome [3]. The mature CCV supporting pathogen replication is a specialized compartment that engages autophagic, endocytic, and secretory pathways [4]. CCV biogenesis and successful infection requires manipulation of several host cell functions by effector proteins secreted by a specialized Dot/Icm type IV secretion system (T4BSS) [4].

Central to *C*. *burnetii* pathogenesis is resistance to, or avoidance of, macrophage innate immune responses. Inhibitory components of innate immunity include reactive nitrogen intermediates and oxygen species, antimicrobial peptides, nutrient limitation, autophagy, and programmed cell death [5, 6]. The antagonistic effects of several of these factors/processes are associated with fusion of lysosomes with compartmentalized bacteria, a process promoted by autophagic machinery [7]. However, *C*. *burnetii* is naturally resistant to lysosomal activities of resting macrophages. In fact, the T4BSS of *C*. *burnetii* mediates interactions between the CCV and the autophagy pathway that appear to benefit CCV formation and/or pathogen growth [8–11]. Infection also renders host cells resistant to inducers of apoptosis and pyroptosis, with four *C*. *burnetii* T4BSS effector proteins known to exert pro-survival effects [12–14].

Induction of cell autonomous innate immune functions requires detection of bacterial pathogen-associated molecular patterns (PAMPs) by pathogen-recognition receptors (PRR) [15]. Toll-like receptors (TLR) of the plasma membrane primarily detect bacterial surface PAMPs while cytosolic PRRs detect PAMPs released into the cytoplasm. Typically, PAMP recognition results in classically (M1) activated macrophages with pro-inflammatory properties that thwart invading bacterial pathogens [16, 17]. However, some pathogens are adept at preventing M1 polarization or promoting polarization of alternatively (M2) activated macrophages, which are considered anti-inflammatory [17].

The degree and outcome of PRR signaling triggered by *C*. *burnetii* infection primarily depends on lipopolysaccharide (LPS) length [18, 19]. Natural isolates of *C*. *burnetii* that produce a full-length LPS undergo a serologically-defined phase I (virulent) to phase II (avirulent) transition after extensive *in vitro* passage. Phase II bacteria produce a severely truncated LPS containing lipid A and some core sugars, but no *O*-antigen [20–22]. Interactions between isogenic *C*. *burnetii* Nine Mile phase I (NMI) and phase II (NMII) reference strains and cultured myeloid cells have been extensively characterized [23]. The *O*-antigen of NMI appears to mask the outer membrane and limit recognition of surface PAMPs, such as lipoproteins [18, 19, 24]. Consequently, relative to *in vitro* infection by NMII, NMI induces a limited pro-inflammatory response. NMI and NMII produce an identical tetra-acylated lipid A that weakly interacts with TLR 4 [25].

Induction of a potentiated innate immune response by NMII is considered an important mechanism that clears the organism from an immunocompetent animal in the absence of disease [19, 26, 27]. Relative to NMI, *in vitro* infection of primary human myeloid cells by NMII results in production of more pro-inflammatory cytokines, such as tumor necrosis factor (TNF), interleukin-12p70 (IL-12p70) and interleukin-1ß (IL-1ß), but ultimately both strains replicate similarly [3, 19, 28]. In contrast, the robust pro-inflammatory response observed upon NMII infection of murine primary bone marrow-derived macrophages (BMDM) is associated with moderate to severe growth restriction, depending on the mouse strain [25, 27, 29–35]. For example, BMDM from A/J and Balb/c mice are moderately permissive for NMII growth while macrophages from C57BL/6 are highly restrictive [30]. A recent comprehensive study of NMII engagement of the innate immune response of C57BL/6 BMDM confirmed the importance of TLR2 in inhibiting NMII growth through production of TNF [27]. No role for cytosolic PRRs was established [27]. These results are in accordance with previous studies showing BMDM from TLR2 knockout mice are markedly more permissive for growth of NMII than wild type macrophages [25], and that the corresponding knockout mouse is more susceptible to NMII infection [36]. Nitric oxide appears to be a key inhibitory effector induced by TNF [27]. Indeed, nitric oxide has been previously shown to inhibit NMII replication in murine L-929 fibroblasts [37] and BMDM [31, 32].

To our knowledge, conditioned medium from murine L-929 cell cultures has been used in published NMII infection studies as the source of macrophage-colony stimulating factor (M-CSF) for differentiation of bone marrow-derived myeloid progenitors into macrophages [25, 27, 29–35]. Here, we demonstrate that BMDM from C57BL/6J mice are highly permissive for growth of NMII when myeloid progenitor cells are differentiated with recombinant murine M-CSF (rmM-CSF). Bacteria achieve 2 to 3 logs of growth over 6 days in phagolysosome-like vacuoles. Both infected and uninfected BMDM lacked defined M1/M2 polarization, and infection resulted in production of moderate amounts of TNF and nitric oxide. Similar robust growth was also observed in macrophages obtained from wild type and natural resistance-associated macrophage protein 1 (Nramp1) knock-in C57BL/6J mice, where macrophages were derived from myeloid progenitors immortalized with an estrogen-regulated *Hoxb8* (*ER-Hoxb8*) oncogene. Collectively, our results show that primary and immortalized macrophages from C57BL/6J mice can be used to define host and bacterial determinants involved in productive infection of murine macrophages by avirulent phase II *C*. *burnetii*.

## Materials and methods

### Bacteria

*C*. *burnetii* Nine Mile RSA439 (phase II, clone 4) was utilized throughout this work and was grown microaerobically in ACCM-2 as previously described [38]. *Salmonella enterica* serovar Typhimurium strain SL1344 was grown in Luria-Bertani (LB) Miller media plus streptomycin (100 μg/ml) at 37°C with aeration at 225 rpm [39].

### Culture and infection of BMDM

BMDM from C57BL/6J mice were generated as described by Crane *et al*. [40] with minor modifications. Female 8 to 12-week old C57BL/6J mice were sacrificed by deep anesthesia with isoflurane followed by cervical dislocation. Progenitor cells were isolated from femurs and tibia, and cultured in Dulbecco’s modified Eagle’s Medium (DMEM) + GlutaMAX (Gibco) containing 10% heat-inactivated fetal bovine serum (FBS), 1 mM sodium pyruvate (Corning), 20 ng/ml rmM-CSF (Peprotech), and penicillin/streptomycin (pen/strep) (Thermo Fisher Scientific) in 75-cm^2^ flasks. After 2 days of incubation at 37°C in 5% CO_2_, non-adherent cells were collected and sub-cultured with fresh medium lacking pen/strep (BMDM growth medium) into new 75-cm^2^ flasks. Adherent cells were collected after 4 days and replated in 24 or 6-well plates in fresh medium containing rmM-CSF. Cells were cultivated overnight for respective experiments as detailed below. For infection, the medium was removed and the cells infected with NMII at the desired multiplicity of infection (MOI), based on genome equivalents, in a volume of 250 μl (24-well plates) or 2 ml (6-well plates) BMDM growth medium. Plates were centrifuged at 500 x *g* for 25 min at 37°C. BMDM were washed twice with 1 ml phosphate-buffered saline (PBS; 1.5 mM KH_2_PO_4_, 2.7 mM Na_2_HPO_4_-7H_2_0, 155.0 mM NaCl, pH 7.2), and then 1 ml of BMDM growth medium was added to each well. For differentiation with L-929 cell-conditioned medium, rmM-CSF was replaced with 10% conditioned medium in BMDM growth medium.

### Generation of ER-Hoxb8 progenitor cell lines

Myeloid progenitors from C57BL/6J and C57BL/6J *Nramp1*^*+*^ mice [41] were conditionally immortalized with an estrogen-regulated *Hoxb8* (*ER-Hoxb8*) oncogene [42]. To generate retrovirus expressing the ER-Hoxb8 fusion, pHA-Hoxb8-MSCV-Neo and pEcoPac (both generously provided by Mark Kamps, University of California-San Diego) were co-transfected into 293T human kidney cells (ATCC CRL-3216) using a CaPO_4_ transfection kit (Invitrogen) as described [42]. Transfected cells were cultured in DMEM + GlutaMAX, 10% FBS, and pen/strep (Thermo Fisher Scientific) at 37°C in 5% CO_2_. Culture supernatants containing packaged retrovirus were harvested at 3 and 4 days post-transfection and stored at -80°C. Titers of retrovirus stocks were determined by infecting NIH3T3 murine fibroblasts (ATCC CRL-1658) with 10-fold dilutions of virus and quantifying the number of neomycin-resistant colony forming units observed after 2 weeks of culture in the presence of 1 mg/ml geneticin (Thermo Fisher Scientific).

Immortalized macrophage progenitor cells were generated as described by Wang *et al*. [42]. Briefly, bone marrow was harvested from mouse femurs and myeloid progenitor cells enriched using a mouse hematopoietic Progenitor Cell Isolation Kit (StemCell Technologies, Vancouver, British Columbia). Progenitor cells were cultured in stem cell medium (RPMI + GlutaMAX, 15% FBS, pen/strep [Thermo Fisher Scientific], 10 ng/ml murine IL-3, 20 ng/ml murine IL-6, and 30 ng/ml murine stem cell factor [Peprotech]) at 37°C in 5% CO_2_. After 4 days of incubation, expanded progenitor cells (2.5 x 10^5^) were added to 250 μl of stem cell medium with Lipofectamine (Thermo Fisher Scientific), then mixed with 1 ml of *Hoxb8-ER* retrovirus (>2 x 10^6^ colony forming units per ml) in wells of a fibronectin-coated 12-well tissue culture plate. Plates were centrifuged for 90 min at 1,500 x *g*, then 3 ml of Mac-Pro medium (RPMI + GlutaMAX, 10% FBS, pen/strep, 20 ng/ml murine granulocyte-macrophage colony-stimulating factor [GM-CSF, Peprotech], 30 μM beta-mercaptoethanol, and 1 μM beta-estradiol [Sigma]) were added to each well and the plate was incubated at 37°C in 5% CO_2_. Non-adherent cells were split into fresh Mac-Pro Medium every 3 to 4 days. By 2 weeks post-transduction, round macrophage progenitor cells in non-adherent or loosely adherent clusters were proliferating in wells, whereas no viable cells were found in non-transduced control wells.

### Differentiation of progenitor cell lines into macrophages

Non-adherent ER-Hoxb8 progenitor cells were harvested by centrifugation and washed twice with PBS to remove beta-estradiol. The cells were then resuspended in Mac-Diff medium (RPMI + GlutaMax, 10% FBS, and 20 ng/ml rmM-CSF without beta-estradiol), seeded in 75-cm^2^ flasks, and cultured for 6 days at 37°C in 5% CO_2_. Cells were harvested by replacing the medium with cold PBS, chilling the flask on ice for 20 to 30 min, gently scraping the flask with a sterile cell scraper (Corning), and then centrifuging the cells for 5 min at 500 x *g*. Macrophages were seeded in a 24-well plate (2 x 10^5^ cells per well) in Mac-Diff medium for infection studies.

### Quantification of NMII growth and *Salmonella* survival

Macrophages in 24-well plates (2 x 10^5^ cells per well) were infected with *C*. *burnetii* as described above. To harvest cells for quantitative PCR (qPCR), cells were scraped into growth medium and the suspension transferred to a 1.5 ml centrifuge tube. Wells were washed with 250 μl PBS, which was added to the centrifuge tube. Cells were harvested daily with the sample taken immediately after infection considered 0 days post-infection. Cells and bacteria were pelleted by centrifugation at 20,000 x *g* for 15 min. Supernatants were aspirated and pellets stored at -20°C until processing for qPCR of NMII genome equivalents (GE). Pellets were suspended in 200 μl of nuclease-free water and the suspension transferred to a 2 ml tube containing approximately 50 μl of 0.1 mm Zirconia/silica beads (BioSpec). Two cycles of bead beating were performed on each sample at a power of 5 for 40 sec using a ThermoElectron FastPrep FP120. Samples were heated at 99°C for 10 min and subjected to TaqMan qPCR using a StepOnePlus Real-Time PCR system (Applied Biosystems) to determine the copy number of *C*. *burnetii dotA* [43].

For infection of macrophages with *Salmonella* Typhimurium, bacteria were grown for 18 h to stationary phase. BMDM in 24-well plates (2 x 10^5^ cells per well) were infected at an MOI of 5 by centrifuging bacteria, suspended in 500 μl of BMDM growth medium, onto monolayers at 1000 x *g* for 10 min. At 30 min post-infection, the cells were washed once with PBS and BMDM growth medium with gentamicin (50 μg/ml) was added to each well. Media was replaced at 1.5 h post-infection with media containing 10 μg/ml gentamicin. Incubations were conducted at 37°C in 5% CO_2_ and media was prewarmed to 37°C prior to use. Colony-forming units (CFUs) were collected at 1, 2, and 10 h post-infection following lysis of macrophages with 0.2% sodium deoxycholate in PBS. Serial dilutions were made and plated on LB-agar plates.

### Flow cytometry

BMDM (6 x 10^5^ cells per well) in 6-well plates were infected as above with NMII at an MOI of 10 or left uninfected. At 56 h post-infection, cells were mock treated or treated with 100 ng/ml *Escherichia coli* 0111:B4 lipopolysaccharide (LPS; Sigma) or 20 ng/ml murine IL4 (R & D Systems). Six h prior to treatment with LPS, cells were also treated with 20 ng/ml murine IFN**γ** (R & D Systems). At 72 h post-infection, cells were pooled based on treatment and a minimum of 5 x 10^5^ cells was aliquoted into a 96-well round bottom plate or polystyrene tubes. Cells were washed and suspended in serum-free PBS, then stained with Fixable Viability Dye eFluor 780 according to the manufacturer’s protocol (eBioscience). Cells were suspended in staining buffer (PBS + 2% FBS) containing anti-mouse CD16/32 antibody (Clone 2.4G2; BD Biosciences) for blocking of F_c_ receptors to minimize nonspecific binding. Cells were then suspended in staining buffer containing a cocktail of fluorochrome-conjugated antibodies specific for cell surface antigens including: CD11b (Manufacturer: eBioscience, Clone: M1/70, Fluorophore: eFluor 450), F4/80 (eBioscience, BM8, Brilliant Violet 605), and CD38 (eBioscience, 90, Phycoerythrin). Following surface staining, cells were washed in staining buffer and fixed using the Foxp3/Transcription Factor Staining Buffer Set (eBioscience). Following fixation, cells were permeabilized using the Foxp3/Transcription Factor Staining Buffer Set and suspended in permeabilization buffer containing a cocktail of fluorochrome-conjugated antibodies specific for intracellular antigens including: Egr2 (eBioscience, erongr2, Allophycocyanin) and NMII (generated in-house, rabbit anti-NMII, unconjugated). Cells were washed in permeabilization buffer and suspended in this buffer containing goat anti-rabbit Alexa Fluor 488 IgG (Thermo Fisher Scientific) to stain NMII. Following intracellular staining, cells were suspended in staining buffer and analyzed on an LSR II flow cytometer using FACSDiva software (BD Biosciences). A minimum of 5 x10^4^ events was captured for each sample. Following gating of all cells (FSC-A/SSC-A), live macrophages (Fixable Viability Dye eFluor 780) were first identified by their expression of CD11b and F4/80, then infected cells were identified based on NMII signal. M1 and M2 polarized subsets were identified based on CD38 and Egr2 expression, respectively [16, 44]. Isotype staining controls were included to help set gating boundaries and to ensure specificity of staining. Data analysis was performed with FlowJo v10.1 software (TreeStar Inc., Ashland, OR).

### Nitrite and TNF measurements

BMDM (2 x 10^5^ cells per well) in 24-well plates were infected with NMII at an MOI of 50. At 8 h post-infection, cells were mock treated or treated with 100 ng/ml LPS or 10 μg/ml zymosan (Sigma). For nitrite measurements, cells were also treated with 10 ng/ml murine TNF (PeproTech). At 24 h post-infection, culture supernatants were assayed for the relative level of nitric oxide by determining nitrite concentration with a Griess Reagent System (Promega). Culture supernatants stored at -20°C were assayed for TNF concentrations using a TNF ELISA kit (R & D Systems).

### Confocal fluorescence microscopy

Primary BMDM and ER-Hoxb8 macrophages (2 x 10^5^ cells per well) seeded on 12-mm coverslips in a 24-well plate were infected with NMII at an MOI of 10 and incubated for the indicated times. For NMII, LAMP1, and DNA staining, cells were fixed for 20 min with 4% parafomaldehyde/PBS, followed by permeabilization and blocking for 30 min with 0.05% saponin/5% FBS/PBS. For NMII and DNA staining, cells were fixed and permeabilized with 100% methanol for 10 min, followed by blocking for 30 min with 5% FBS/PBS. NMII was labeled using rabbit polyclonal serum directed against formalin-fixed bacteria and goat anti-rabbit Alexa Fluor 594 IgG (Thermo Fisher Scientific). LAMP1 was labeled with rat anti-mouse LAMP1 (Santa Cruz Biotechnology) and goat anti-rat Alexa Fluor 488 IgG (Thermo Fisher Scientific). Coverslips were mounted using ProLong Gold containing 4',6-diamidino-2-phenylindole (DAPI; Thermo Fisher Scientific) to visualize nuclei. Cells were viewed by confocal fluorescence microscopy using a Zeiss LSM 710 confocal fluorescence microscope. Images were acquired using ZEN imaging software (Carl Zeiss, Inc.).

The proteolytic activity of vacuoles harboring NMII in BMDM was assessed by degradation of DQ Green BSA. BMDM (1 x 10^4^ cells per well) in an 8-well Ibidi dish (Ibidi, Verona, WI) were infected with NMII at an MOI of 100. At 3 days post-infection, cells were incubated for 4 h in culture medium containing DQ Green BSA (50 μg/ml; Thermo Fisher Scientific) and Hoescht (20 μM). Live cells were imaged by confocal fluorescence microscopy using a Perkin-Elmer UltraView spinning disc confocal system connected to a Nikon Eclipse Ti-E inverted microscope with a LiveCell stage top incubator. Images were acquired using an Andor iXon Ultra EMCCD camera (Andor Technology, Inc., Belfast, United Kingdom) and NIS-Elements imaging software (Nikon Instruments, Inc.). All images were processed with ImageJ software written by W. S. Rasband at the U. S. National Institutes of Health, Bethesda, MD, and available from (https://imagej.nih.gov/ij/).

### Ethics statement animal care and use

All experiments were performed in the Association for Assessment and Accreditation of Laboratory Animal Care-accredited Rocky Mountain Laboratories Animal Facility.

This study (ASP #2016–058) was approved by the Rocky Mountain Laboratories Institutional Animal Care and Use Committee, National Institute of Allergy and Infectious Disease, National Institutes of Health.

## Results

### NMII grows robustly in degradative, lysosome-like vacuoles of C57BL/6J BMDM differentiated with rmM-CSF

**Fig 1:**
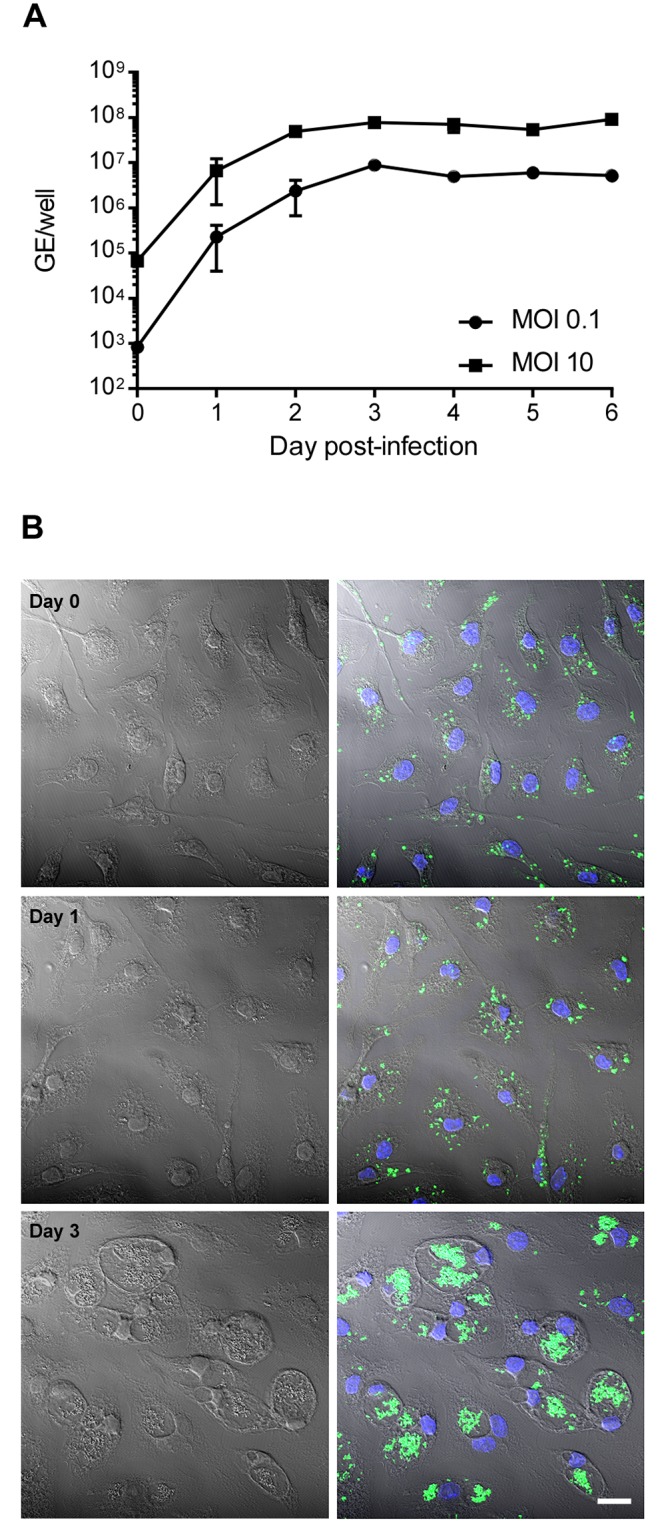
NMII grows robustly in C57BL6/J BMDM differentiated with rmM-CSF. (A) BMDM in 24-well plates were infected with NMII at an MOI of 0.1 or 10. Bacterial growth was assessed by quantifying genomic equivalents (GE). Results are expressed as the means of three biological replicates from one experiment and are representative of three independent experiments. Error bars indicate the standard deviations of the means. (B) BMDM on coverslips in 24-well plates were infected with NMII at an MOI of 10. Cells were fixed immediately after infection (Day 0), and at 1 and 3 days post-infection. Cells were stained for NMII (green) and DNA (blue), and fluorescent images overlaid with the corresponding phase contrast image. Bar, 10 μm.

Previous studies using C57BL/6 BMDM differentiated with L-929 cell-conditioned medium show a severe growth restriction of NMII [25, 27, 30–35]. We therefore tested NMII growth in BMDM differentiated with rmM-CSF because this recombinant growth factor is recommended for experimental reproducibility [44]. BMDM were infected with NMII at MOIs of 0.1 and 10, and bacterial growth was monitored daily for 6 days by qPCR of GE. Roughly 3 log increases in NMII GE were observed over the infection time course (Fig 1A). By immunofluorescence microscopy, increases in genome numbers correlated with proliferation of *C*. *burnetii* within macrophages (Fig 1B). These micrographs also demonstrated initial infection efficiencies near 100%. As a control, we also infected BMDM differentiated with L-929 cell-conditioned medium. At 5 days post-infection, large *Coxiella*-containing vacuoles were evident in macrophages treated with rmM-CSF, but not L-929 cell-conditioned medium (S1 Fig).

**Fig 2:**
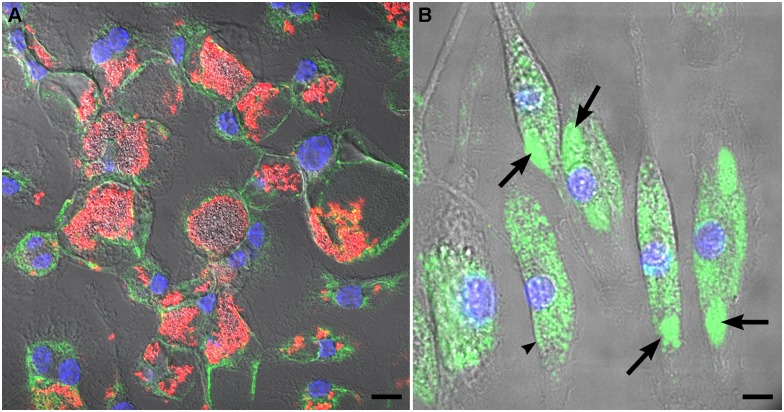
The NMII-occupied vacuole of C57BL/6J BMDM has lysosomal characteristics. (A) BMDM infected for 3 days and stained for NMII (red), LAMP1 (green), and DNA (blue). (B) BMDM infected for 3 days and incubated with DQ Green BSA to detect lysosomal proteolytic activity (green). Arrows denote *Coxiella*-containing vacuoles. An arrowhead demarks an uninfected cell. In both panels, nuclei are stained with Hoescht (blue) and fluorescent images are overlaid with the corresponding phase contrast image. Bars, 5 μm.

Cumulative data from several cell culture model systems indicate that *C*. *burnetii* replicates in vacuoles with lysosomal characteristics [4]. To evaluate the nature of vacuoles harboring replicating NMII in C57BL/6J BMDM, infected cells were stained by immunofluorescence for LAMP1 or loaded with DQ Green BSA, a fluorogenic substrate where fluorescence is quenched until hydrolysis by lysosomal proteases [3]. Large and spacious vacuoles containing abundant NMII were clearly labeled with LAMP1 in BMDMs infected for 3 days (Fig 2A). *Coxiella*-containing vacuoles also exhibited pronounced labeling with DQ Green BSA, confirming their lysosomal nature (Fig 2B).

### C57BL/6J BMDM infected by NMII lack defined polarization

**Fig 3:**
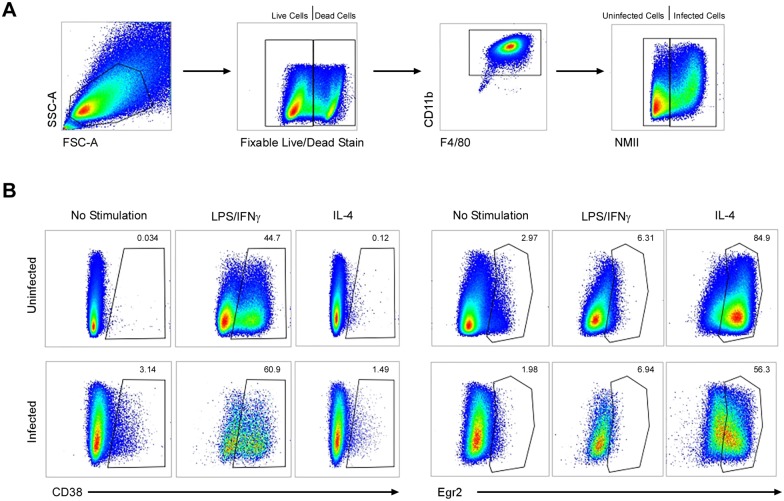
Permissive C57BL/6J BMDM display an M0 phenotype. BMDM were mock infected or infected with NMII at an MOI of 10. At 56 h post-infection, cells were treated with medium (no stimulation), 100 ng/ml LPS, or 20 ng/ml IL4. Six h prior to treatment with LPS, cells were also treated with 20 ng/ml IFN**γ**. Flow cytometry was conducted at 72 h post-infection. (A) Cells were gated on live CD11b^+^ F4/80^+^ cells with additional gating on infected cells based on NMII signal. (B) Expression of CD38 and early growth response protein 2 (Egr2), markers of classically activated (M1) and alternatively activated (M2) macrophages, respectively. Results are representative of three independent experiments.

**Fig 4:**
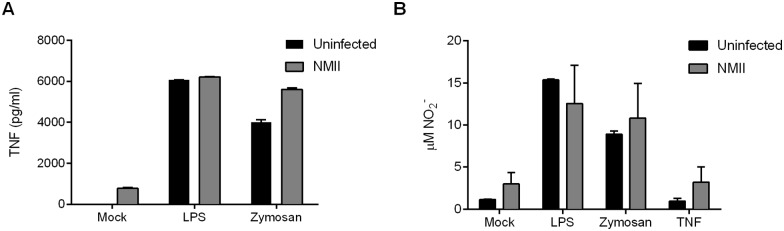
NMII-infected C57BL/6J BMDM produce moderate amounts of TNF and nitric oxide. BMDM were infected with NMII at an MOI of 50. At 8 h post-infection, cells were mock treated or treated with 100 ng/ml LPS (TLR4 ligand), 10 μg/ml zymosan (TLR2 ligand), or 10 ng/ml TNF (nitrite assay only). Following an 18 h treatment, culture supernatants were assayed for TNF by using an ELISA kit (A) and nitric oxide by determining nitrite concentration with the Griess reagent (B). Results are expressed as the means of three biological replicates from one experiment and are representative of two independent experiments. Error bars indicate the standard deviations of the means.

Because macrophage activation status is associated with restriction of infection by bacterial pathogens, including *C*. *burnetii* [17], we examined the expression of M1/M2 polarization markers [16, 44] by flow cytometry. Live, mock infected BMDM (F4/80^+^ CD11b^+^), showed no appreciable expression of the M1 and M2 markers CD38 and early growth response protein 2 (Egr2), respectively (Fig 3A and 3B). Infection by NMII for 3 days did not change this M0 (non-polarized) phenotype (Fig 3A and 3B). Similar results were obtained at 1 day post-infection (data not shown). Infected and uninfected BMDM treated with LPS/IFN**γ** and IL-4 showed pronounced induction of CD38 and Egr2, respectively, thereby demonstrating appropriate M1 and M2 responses, respectively, to each stimulus (Fig 3B). We further assessed BMDM responses during infection by measuring levels of secreted TNF and production of nitric oxide (as measured by medium nitrite levels). In comparison to uninfected BMDM, NMII-infected cells produced slightly more TNF and nitric oxide (Fig 4A and 4B). Levels did not approach those associated with infected or uninfected BMDM treated with LPS, zymosan, or TNF (nitric oxide measurements only). Overall, these data demonstrate that uninfected and NMII-infected murine BMDM differentiated with rmM-CSF display an M0 phenotype. Moreover, infection does not inhibit M1 or M2 polarization in the presence of appropriate stimuli.

### Wild type and Nramp1-positive ER-Hoxb8 macrophages are permissive for NMII growth

**Fig 5:**
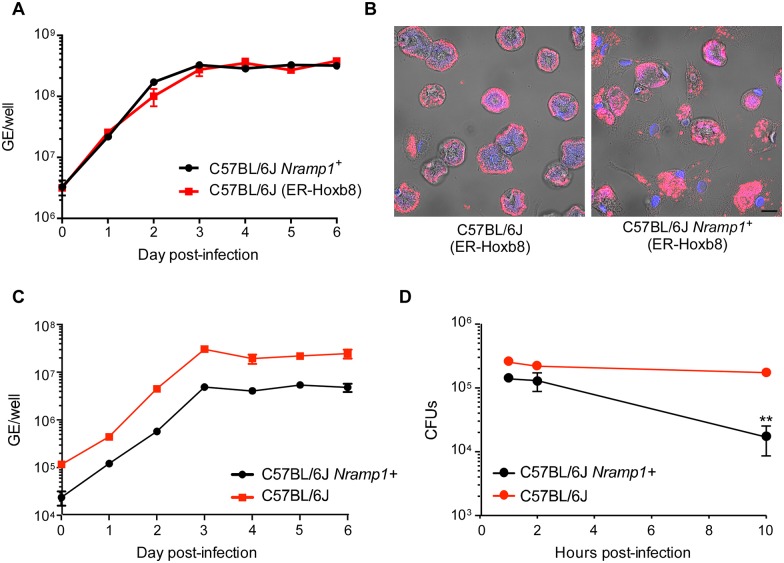
Wild type and Nramp1-positive ER-Hoxb8 macrophages are permissive for NMII growth. (A) Bone marrow-derived myeloid progenitors from wild type and C57BL/6J *Nramp1+* mice were immortalized with an estrogen-regulated *Hoxb8* (*ER-Hoxb8*) oncogene. Macrophages were derived from immortalized progenitors using M-CSF and infected with NMII at an MOI of 10. Approximately 2 logs of NMII growth, based on genome equivalents (GE), was observed in both C57BL/6J and C57BL/6J ER-Hoxb8 *Nramp1+* macrophages over a 6 day time course. Results are expressed as the means of three biological replicates from one experiment and are representative of two independent experiments. Error bars indicate the standard deviations of the means. (B) Confocal fluorescence micrographs of ER-Hoxb8 micrographs showing large vacuoles harboring NMII (red) in both cell types at 3 days post-infection. DNA is stained with DAPI (blue). Bar, 10 μm. (C) Primary BMDM from wild type and C57BL/6J *Nramp1+* mice differentiated with rmM-CSF were infected with NMII at an MOI of 0.1. Approximately 2 logs of NMII growth, based on genome equivalents (GE), was achieved at early stationary phase (3 days post-infection) in both cell types. (D) Primary BMDM from wild type and C57BL/6J *Nramp1+* mice differentiated with rmM-CSF were infected with *Salmonella* Typhimurium at an MOI of 5. Colony forming units (CFUs), representing intracellular bacteria, were enumerated at 1, 2, and 10 h post-infection by plating macrophage lysates on LB-agar plates. Significantly less CFU were recovered from *Nramp1+* macrophages then from wild type macrophages at 10 h post-infection (asterisks, *p* <0.01). Results are expressed as the means of three biological replicates from one experiment and are representative of two independent experiments. Error bars indicate the standard deviations of the means.

Our data clearly show that primary BMDM from C57BL/6J mice can support robust growth of NMII. An important implication of this finding is that genetic determinants of host susceptibility to NMII infection can now be evaluated using the vast array of knock-in, transgenic, and knock-out mice available in a C57BL/6 genetic background [45]. However, use of primary BMDM as a model system requires time-consuming preparation protocols and maintenance of breeding colonies. We therefore examined whether macrophages obtained from bone marrow-derived myeloid progenitors of C57BL/6J mice immortalized with an estrogen-regulated *Hoxb8* (*ER-Hoxb8*) oncogene could support NMII growth [42]. In this system, myeloid progenitors expressing ER-Hoxb8 are continually renewed when cultured in the presence of beta-estradiol. Removal of estrogen and culture in rmM-CSF results in differentiation of progenitors into macrophages [46]. ER-Hoxb8 macrophages supported approximately 2 logs of growth of NMII prior to entry into stationary phase (Fig 5A). Replication corresponded to cells exhibiting large NMII-filled vacuoles (Fig 5B).

We extended our evaluation of the ER-Hoxb8 system to macrophages derived from myeloid progenitors of natural resistance-associated macrophage protein 1 (Nramp1) C57BL/6J transgenic mice. Nramp1 is a phagosome-associated metal transporter that depletes pathogen-containing vacuoles of divalent metals, such as Fe^2+^ and Mn^2+^ [47]. This component of nutritional immunity [47, 48] can restrict infection by intracellular pathogens that reside in vacuoles of the endolysosomal system [49]. Wild type C57BL/6J mice have a Gly169Asp substitution that results in defective Nramp1 production [47]. Replication and vacuole formation by NMII in wild type and ER-Hoxb8 *Nramp1*^*+*^ macrophages were indistinguishable (Fig 5A and 5B), suggesting Nramp-mediated metal depletion of the CCV does not negatively impact NMII growth. To validate our results with ER-Hoxb8 macrophages, we infected primary BMDM from wild type and C57BL/6J *Nramp1+* mice differentiated with rmM-CSF with NMII and *Salmonella enterica* serovar Typhimurium. In agreement with ER-Hoxb8 macrophage results, no defect in NMII growth was detected in *Nramp1+* primary BMDM relative to wild type macrophages (Fig 5C). In contrast, and in keeping with several published studies showing an *in vivo* role for Nramp1 in controlling *Salmonella* infection [41, 50–52], a significant decline in recoverable CFUs was detected from 6 to 10 h post-infection in *Nramp1+* BMDM relative to wild type macrophages (Fig 5D).

## Discussion

Depending on the strain of origin, primary murine BMDM have been previously reported to be moderately to markedly restrictive for NMII growth [25, 27, 29–34]. The biological basis of this behavior has been investigated in detail using highly restrictive BMDM from C57BL/6 mice. A working model attributes restriction of NMII to increased PAMP exposure that elicits production of inhibitory amounts of TNF and nitric oxide [23, 25, 27, 30–34]. Indeed, Bradley *et al*. [27] recently elucidated the importance of TNF by showing infection induces production of the cytokine, and that growth permissiveness of *Tnf* ^*-/-*^ macrophages is reversed by adding recombinant TNF or medium from infected C57BL/6 BMDM cultures. Furthermore, infected *Tnf* ^*-/-*^ BMDM produce lower levels of nitric oxide than wild type cells, invoking a potential inhibitory role for this innate immune mediator. To our knowledge, conditioned medium from murine L-929 cell cultures has been exclusively used in published NMII BMDM infection studies as a source of M-CSF to differentiate bone marrow-derived myeloid progenitors into macrophages. Here, we show that bone marrow progenitors of C57BL/6J mice differentiated with rmM-CSF are highly permissive for NMII growth, a phenotype that correlates with the lack of defined M1/M2 polarization. Interestingly, using similar infection conditions and time points, culture supernatant levels of nitrite in our study and the Bradley *et al*. study [27] were roughly the same (~2 μM) while TNF levels were approximately 3 times higher in the latter. These results suggest nitric oxide may play a minor role in limiting growth of NMII under restrictive BMDM culture conditions while additional TNF-induced factors play more substantial roles. Furthermore, BMDM cultured with L-929 cell-conditioned medium versus rmM-CSF may be primed to produce more TNF that limits NMII growth via activation of downstream effectors.

The 6 day stationary phase growth yields (2 to 3 logs) of NMII in C57BL/6J BMDM are similar to other cell culture infection systems, such as Vero epithelial cells and THP-1 macrophages [43, 53–55]. Early stationary phase was consistently reached no later than 3 days post-infection; however, in some growth curves NMII grew with accelerated lag/log phase kinetics relative to other host cell systems [43, 53]. The modest curve-to-curve variability in early time point growth kinetics may be due to several parameters including method of infection (e. g., centrifugation of inoculum or not), stock viability, DNA yield, inherent variability in qPCR (especially at early time points with low DNA concentrations), and MOI. From axenic media studies, we know that NMII is capable of a generation time much shorter then 10 to 11 h measured in the Vero cell system (4.7 h in ACCM-2) [38, 43]. Infections at MOIs >1 also generally have abbreviated lag/log phases, which we attribute to homotypic fusion of multiple *Coxiella*-containing vacuoles, a process that accelerates development of a replication-competent niche. Our infection procedure incorporated a centrifugation step, which aids internalization. Indeed, an infection efficiency approaching 100% was achieved using an MOI of 10. Another factor potentially contributing to shortened lag phase is faster acidification of the NMII vacuole in BMDM than epithelial cells. A pH of approximately 5 is required for vigorous metabolic activation of *C*. *burnetii* and commencement of replication [56]. Indeed, Newton and co-workers [35] demonstrated translocation of NMII effector proteins as early as 1 h post-infection in BMDM as compared to 8 h post-infection in HeLa cells. Effector translocation is an energy requiring process and a predicted signature of metabolic activation [53].

Methods used to isolate, cultivate, and infect BMDM can dramatically affect their polarization state and permissiveness for microbial infection [44]. Growth parameters including the method of bone marrow processing, cell density, culture conditions, and media supplements all impact macrophage development [44, 57]. Conditioned medium from murine L-929 cell cultures is commonly used as a source of M-CSF to differentiate bone marrow-derived myeloid progenitors into macrophages. However, variation in M-CSF levels in conditioned medium and the presence of type I interferons can affect macrophage responses to invading pathogens [44, 58]. To achieve reproducibility in BMDM experiments between different laboratories, differentiation of progenitors with endotoxin-free, rmM-CSF is now the accepted experimental standard [44]. Moreover, a detailed description of methodology is also recommended [44].

Interestingly, a recent study by Fernandes *et al*. [34] demonstrated that alveolar macrophages from C57BL/6 mice support growth of NMII with 2 logs of replication achieved at 12 days post-infection. Growth permissiveness in fully differentiated alveolar macrophages is attributed to a pronounced M2 polarization as compared to the M1 polarization of control BMDMs that were differentiated from monocytes with L-929 cell-conditioned medium. In general, alveolar macrophages are considered less pro-inflammatory than other tissue-resident macrophages, a property that enables environmental sampling without damaging immune responses [59]. Although the NMII growth yield per macrophage is roughly the same between the alveolar macrophages described by Fernandes *et al*. [34] and the BMDM of this report, bone marrow yields approximately 100-fold more macrophages than lungs (7 x 10^7^ versus 4 x 10^5^ per mouse, respectively), which greatly facilitates functional assays [60]. Nonetheless, alveolar macrophages are the initial target cell of aerosol-transmitted *C*. *burnetii* and consequently may constitute a more physiologically relevant *ex vivo* model system [61].

Nramp1 expression is associated with reduced animal model pathogenesis in the context of infection by *Salmonella*, *Leishmania*, and some species of *Mycobacteria* [50, 62]. Consistent with *in vivo* studies [41, 50–52], here we demonstrated that BMDM from C57BL/6J *Nramp1+* knock-in mice more readily kill *Salmonella* Typhimurium than wild type macrophages. In contrast, NMII shows no growth deficiency in *Nramp1+* primary BMDM or immortalized ER-Hoxb8 macrophages derived from C57BL/6J mice, suggesting Nramp1 removal of metal ions from the CCV does not have an inhibitory effect, at least during *in vitro* growth. Our results do not discount a protective role for Nramp1 during animal infection. Indeed, patients with post-Q fever fatigue syndrome show an increased frequency of allelic variants of *Nramp1* [63]. Conversely, there is no correlation between the expression of *Nramp1* and the sensitivity of various inbred mouse strains to *C*. *burnetii* infection [64]. Several bacterial pathogens express Nramp1 homologs termed MntH that may compete for metal ions depleted by Nramp1 [49]. However, *C*. *burnetii* does not encode an MntH protein [65]. In fact, the pathogen has few iron acquisition systems, one of which is the metal binding lipoprotein LimB [66]. Taken together with a small Fur regulon and enhanced growth in low iron environments, Briggs *et al*. [67] suggested that *C*. *burnetii* has a low iron requirement and that iron may be actually detrimental to intracellular growth of *C*. *burnetii* by promoting Fenton chemistry that produces damaging hydroxyl radicals [67].

In summary, our results show primary macrophages from wild type and genetically altered C57BL/6J mice can be used to define host-pathogen interactions that promote productive infection by attenuated phase II *C*. *burnetii*.

## Supporting information

S1 Fig C57BL/6J BMDM differentiated with L-929 cell-conditioned medium are less permissive for NMII growth than cells differentiated with rmM-CSF. BMDM infected for 5 days (MOI = 10) were fixed and stained by immunofluorescence for NMII (red). Nuclei (blue) were stained with DAPI. Large *Coxiella*-containing vacuoles are evident in rmM-CSF, but not L-929 cell-conditioned medium treated cells. Bar, 5 μm.
(PDF)
