# Supplementary material for: Robust growth of avirulent phase II Coxiella burnetii in bone marrow-derived murine macrophages
Source: PLoS One. 2017 Mar 9;12(3):e0173528. doi: 10.1371/journal.pone.0173528 (PMC5344453; doi:10.1371/journal.pone.0173528)
Supplement: S1 Fig — BMDM infected for 5 days (MOI = 10) were fixed and stained by immunofluorescence for NMII (red). Nuclei (blue) were stained with DAPI. Large Coxiella-containing vacuoles are evident in rmM-CSF, but not L-929 cell-conditioned medium treated cells. Bar, 5 μm. (PDF) [file pone.0173528.s001.pdf]

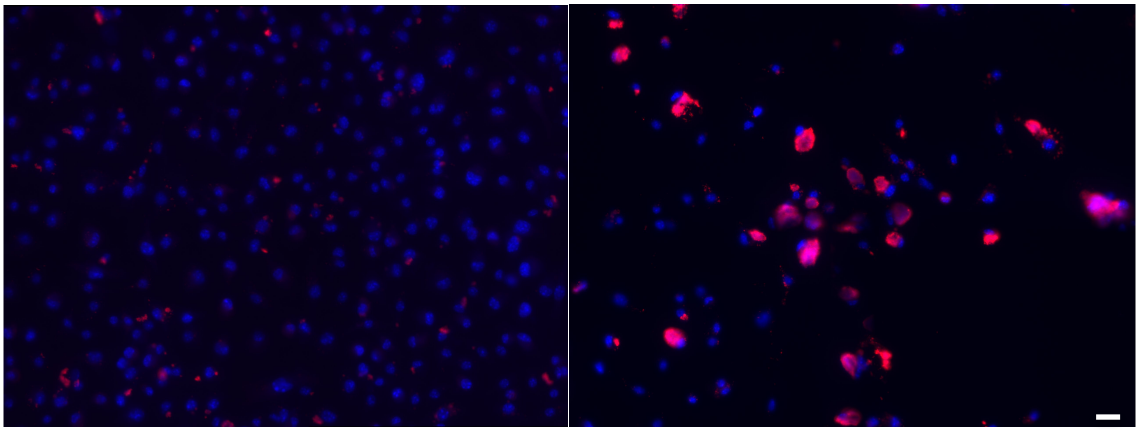

S1 Figure. **BMDM differentiated with L-929 cell conditioned medium are less permissive for NMII growth than cells differentiated with rmM-CSF.** BMDM infected for 5 days (MOI=10) were fixed and stained by immunofluorescence for NMII (red). Nuclei (blue) are stained with Hoescht. Large *Coxiella*-containing vacuoles are evident in rmM-CSF, but not L-929 cell conditioned medium, treated cells. Bar, 10 μm.
